# Influence of elevated-CRP level-related polymorphisms in non-rheumatic Caucasians on the risk of subclinical atherosclerosis and cardiovascular disease in rheumatoid arthritis

**DOI:** 10.1038/srep31979

**Published:** 2016-08-18

**Authors:** Raquel López-Mejías, Fernanda Genre, Sara Remuzgo-Martínez, Carlos González-Juanatey, Montserrat Robustillo-Villarino, Javier Llorca, Alfonso Corrales, Esther Vicente, José A. Miranda-Filloy, César Magro, Beatriz Tejera-Segura, Marco A. Ramírez Huaranga, Trinitario Pina, Ricardo Blanco, Juan J. Alegre-Sancho, Enrique Raya, Verónica Mijares, Begoña Ubilla, María D. Mínguez Sánchez, Carmen Gómez-Vaquero, Alejandro Balsa, Dora Pascual-Salcedo, Francisco J. López-Longo, Patricia Carreira, Isidoro González-Álvaro, Luis Rodríguez-Rodríguez, Benjamín Fernández-Gutiérrez, Iván Ferraz-Amaro, Santos Castañeda, Javier Martín, Miguel A. González-Gay

**Affiliations:** 1 Epidemiology, Genetics and Atherosclerosis Research Group on Systemic Inflammatory Diseases, Division of Rheumatology, IDIVAL, Santander, Spain; 2 Cardiology Department, Hospital Lucus Augusti, Lugo, Spain; 3 Division of Rheumatology, Hospital Universitario Doctor Peset, Valencia, Spain; 4 Department of Epidemiology and Computational Biology, School of Medicine, University of Cantabria, and CIBER Epidemiología y Salud Pública (CIBERESP), IDIVAL, Santander, Spain; 5 Division of Rheumatology, Hospital Universitario la Princesa, IIS-IPrincesa, Madrid, Spain; 6 Rheumatology Department, Hospital Universitario Lucus Augusti, Lugo, Spain; 7 Division of Rheumatology, Hospital Clínico San Cecilio, Granada, Spain; 8 Rheumatology Division, Hospital Universitario de Canarias, Santa Cruz de Tenerife, Spain; 9 Rheumatology Department, Hospital General Universitario de Ciudad Real, Ciudad Real, Spain; 10 Department of Rheumatology, Hospital Universitario Bellvitge, Barcelona, Spain; 11 Department of Rheumatology, Hospital Universitario La Paz, Madrid, Spain; 12 Department of Rheumatology, Hospital General Universitario Gregorio Marañón, Madrid, Spain; 13 Department of Rheumatology, Hospital Universitario 12 de Octubre, Madrid, Spain; 14 Department of Rheumatology, Hospital Clínico San Carlos, Madrid, Spain; 15 Institute of Parasitology and Biomedicine López-Neyra, IPBLN-CSIC, Granada, Spain; 16 School of Medicine, University of Cantabria, Santander, Spain; 17 Cardiovascular Pathophysiology and Genomics Research Unit, School of Physiology, Faculty of Health Sciences, University of the Witwatersrand, Johannesburg, South Africa

## Abstract

Association between elevated C-reactive protein (CRP) serum levels and subclinical atherosclerosis and cardiovascular (CV) events was described in rheumatoid arthritis (RA). *CRP, HNF1A, LEPR, GCKR, NLRP3, IL1F10, PPP1R3B, ASCL1, HNF4A* and *SALL1* exert an influence on elevated CRP serum levels in non-rheumatic Caucasians. Consequently, we evaluated the potential role of these genes in the development of CV events and subclinical atherosclerosis in RA patients. Three tag *CRP* polymorphisms and *HNF1A, LEPR, GCKR, NLRP3, IL1F10, PPP1R3B, ASCL1, HNF4A* and *SALL1* were genotyped in 2,313 Spanish patients by TaqMan. Subclinical atherosclerosis was determined in 1,298 of them by carotid ultrasonography (by assessment of carotid intima-media thickness-cIMT-and presence/absence of carotid plaques). CRP serum levels at diagnosis and at the time of carotid ultrasonography were measured in 1,662 and 1,193 patients, respectively, by immunoturbidimetry. Interestingly, a relationship between *CRP* and CRP serum levels at diagnosis and at the time of the carotid ultrasonography was disclosed. However, no statistically significant differences were found when *CRP*, *HNF1A, LEPR, GCKR, NLRP3, IL1F10, PPP1R3B, ASCL1, HNF4A* and *SALL1* were evaluated according to the presence/absence of CV events, carotid plaques and cIMT after adjustment. Our results do not confirm an association between these genes and CV disease in RA.

Rheumatoid arthritis (RA) is a chronic disease related to an increased risk of cardiovascular (CV) mortality and high prevalence of subclinical atherosclerosis^1^. Besides traditional CV risk factors^2^ and inflammation^3^, a genetic component appears to be crucial in these processes^2^,^4^,^5^,^6^.

C-reactive protein (CRP) is currently the most widely used biomarker of inflammation^7^. The production of this protein occurs almost exclusively in the liver^8^. Several studies have suggested that elevated serum concentrations of CRP are related to the development of several processes associated with atherosclerosis such as coronary heart disease^9^ and stroke^10^. Accordingly, a relationship between chronic inflammation determined by CRP serum levels and the development of both subclinical atherosclerosis^11^ and CV events^2^ has been found in RA patients.

Genetic variation is a major determinant of CRP levels. In this sense, a meta-analysis has identified several loci associated with elevated CRP serum levels in non-rheumatic Caucasian individuals^12^. *CRP* gene was found to be the most significant signal related to increased CRP serum levels^12^. Other polymorphisms also exert an influence on the elevated level of serum CRP in non-rheumatic Caucasians^12^. It is the case for common genetic variants that play a role in the immune system (*NLRP3* [*NACHT, LRR and PYD domains-containing protein 3*] rs12239046 or *IL1F10* [*interleukin-1 family, member 10*] rs6734238) or in the susceptibility to develop metabolic syndrome (*HNF1A* [*hepatic nuclear factor 1-α*] rs1183910, *LEPR [leptin receptor]* rs4420065, *GCKR* [*glucokinase regulator*] rs1260326 and *HNF4A* [*hepatocyte nuclear factor 4-α*] rs1800961)^12^. A relationship between genetic variants that reside in regions without an apparent role in chronic inflammation (*PPP1R3B* [*protein phosphatase 1, regulatory-inhibitor- subunit 3B*] rs9987289*, SALL1* [*sal-like 1*] rs10521222 and *ASCL1* [*achaete-scute complex homolog 1*] rs10745954) and increased CRP serum levels was also disclosed in non-rheumatic Caucasian individuals^12^.

Taking together all this information, the aim of the present study was to evaluate the potential influence of *CRP, HNF1A, LEPR, GCKR, NLRP3, IL1F10, PPP1R3B, ASCL1, HNF4A* and *SALL1,* polymorphisms related to elevated CRP serum levels in non-rheumatic Caucasians, on the development of CV events and subclinical atherosclerosis in RA patients. For this purpose, we took advantage of data from a large series of RA patients assessed for CV disease.

## Patients and Methodology

### Subjects and Study Protocol

2,313 unrelated patients from Spain were enrolled in our work. Peripheral blood was obtained from subjects recruited from Hospital Universitario Lucus Augusti (Lugo), Marqués de Valdecilla (Santander), Bellvitge (Barcelona), San Cecilio (Granada), Canarias (Tenerife), Doctor Peset (Valencia), General de Ciudad Real (Ciudad Real) and Clínico San Carlos, La Paz, La Princesa, Gregorio Marañón and 12 de Octubre (Madrid).

For experiments involving humans and the use of human blood samples, all the methods were carried out in accordance with the approved guidelines and regulations, according to the Declaration of Helsinki. All experimental protocols were approved by the Ethics Committees of clinical research of Galicia for Hospital Lucus Augusti in Lugo, of Cantabria for Hospital Marqués de Valdecilla in Santander, of Cataluña for Hospital de Bellvitge in Barcelona, of Andalucía for Hospital San Cecilio in Granada, of Canarias for Hospital de Canarias in Tenerife, of Comunidad Valenciana for Hospital Doctor Peset in Valencia, of Castilla-La Mancha for Hospital General de Ciudad Real in Ciudad Real and of Madrid for Hospital Clínico San Carlos, La Paz, La Princesa, Gregorio Marañón and 12 de Octubre in Madrid. Informed consent was obtained from all subjects.

Patients fulfilled the 2010 classification criteria for RA^13^. RA patients were evaluated for *CRP, HNF1A, LEPR, GCKR, NLRP3, IL1F10, PPP1R3B, ASCL1, HNF4A* and *SALL1* polymorphisms. In addition, subclinical atherosclerosis was determined in 1,298 of the RA patients recruited in the study using a carotid ultrasound (US) technique (by evaluation of carotid intima-media thickness -cIMT- and presence/absence of carotid plaques). Also, CRP serum levels at the time of RA diagnosis and at the time of carotid US study were measured in a subgroup of 1,662 and 1,193 patients, respectively, by immunoturbidimetry.

Data on epidemiological and demographical features of the patients recruited in the present work are displayed in Table 1. Traditional CV risk factors and CV events were defined previously^2^,^14^.

### Selection of polymorphisms and genotyping

Since *CRP* has been identified as the most relevant signal related to elevated CRP serum levels in non-rheumatic Caucasians^12^, we performed a tagging of this gene with the aim of covering all its variability. Consequently, we genotyped 3 polymorphisms: *CRP* rs1417938, *CRP* rs1800947 and *CRP* rs1205.

Additionally, we tested 9 genetic variants (*HNF1A* rs1183910*, LEPR* rs4420065*, GCKR* rs1260326*, NLRP3* rs12239046*, IL1F10* rs6734238*, PPP1R3B* rs9987289, *ASCL1* rs10745954, *HNF4A* rs1800961 and *SALL1* rs10521222) previously described as relevant polymorphisms related to increased CRP serum levels in non-rheumatic Caucasians^12^.

Genotyping was performed by TaqMan predesigned assays in a 7900 HT Real-Time polymerase chain reaction system (Applied Biosystems, Foster City, CA, USA).

### US evaluation

Measurement of the cIMT values and presence/absence of carotid plaques were evaluated in 1,298 cases. Patients from Santander, Granada, Tenerife, Valencia, Ciudad Real and Madrid were evaluated by a commercially scanner, Mylab 70, Esaote (Genoa-Italy) as previously described^15^. Patients from Lugo were measured by high-resolution B-mode ultrasound, Hewlett Packard SONOS 5500 as previously reported^16^. cIMT was measured at the far wall of the right and left common carotid arteries over the proximal 15 mm-long segment. cIMT was determined as the average of three measurements in each common carotid artery. Plaque criteria in the accessible extracranial carotid tree (common carotid artery, bulb and internal carotid artery) were focal protrusion in the lumen at least cIMT >1.5 mm, protrusion at least 50% greater than the surrounding cIMT, or arterial lumen encroaching >0.5 mm^17^. The carotid plaques were counted in each territory and defined as no plaque, unilateral plaque or bilateral plaques^17^. Agreement between these two US methods was previously reported^18^. Experts with a high reproducibility and an excellent inter-observer reliability in the evaluation of subclinical atherosclerosis in patients with RA performed the studies.

### Evaluation of CRP serum levels

CRP serum levels at the time of RA diagnosis and at the time of the carotid US study were measured in a subgroup of 1,662 and 1,193 patients, respectively, by an automated immunoturbidimetric assay using the ADVIA Chemistry system (Siemens Healthcare Diagnostics Inc.). Serum samples of RA patients containing CRP form an immune complex with the assay reagent and antibodies against CRP that precipitates, increasing the turbidity of the sample. When light is passed through the reaction solution, some light is absorbed by the precipitates, which is measured at 596/694 nm. The level of CRP is determined by comparison with a calibrator of known concentration.

### Statistical analysis

Genotyping data were checked for deviation from Hardy-Weinberg equilibrium (HWE) using http://ihg.gsf.de/cgi-bin/hw/hwa1.pl. Power for the study was calculated using “CaTS-Power Calculator for Two Stage Association Studies” (http://www.sph.umich.edu/csg/abecasis/CaTS/). Haplotypes were constructed using Haploview v4.2 software.

The relationship between allele/haplotype frequencies and the presence/absence of CV events was tested using Cox regression adjusting for gender, follow-up time, age at RA diagnosis and traditional CV risk factors as confounder factors. Results were expressed as hazard ratios (HR) with 95% confidence interval (CI).

Differences in the allele/haplotype frequencies according to the presence/absence of carotid plaques were calculated by χ^2^ or Fisher tests when necessary (expected values below 5). Strength of associations was estimated using odds ratios (OR) and 95% CI. Results were adjusted for gender, follow-up time, age at the time of US evaluation and traditional CV risk factors as confounder factors by logistic regression.

Association between allele/haplotype frequencies and data on cIMT was evaluated by unpaired t test. Results were adjusted for gender, follow-up time, age at the time of US evaluation and traditional CV risk factors as confounder factors by analysis of covariance (ANCOVA).

Results on CRP serum levels were expressed as mean ± standard deviation (SD). The relationship between allele/haplotype frequencies and CRP serum levels at RA diagnosis and at the time of the carotid US study was evaluated by unpaired t test.

The statistical software used to perform all the statistical analyses was STATA 12/SE (Stata Corp., College Station, TX, USA).

## Results

*CRP* rs1417938, *CRP* rs1800947, *CRP* rs1205*, HNF1A* rs1183910*, LEPR* rs4420065*, GCKR* rs1260326*, NLRP3* rs12239046*, IL1F10* rs6734238*, PPP1R3B* rs9987289*, ASCL1* rs10745954*, HNF4A* rs1800961 and *SALL1* rs10521222 genotype distribution were in HWE (p > 0.05). The genotyping success was greater than 99% in all the cases.

The study had >90% of power to detect genotypic OR = 1.3 for *CRP* rs1417938, *CRP* rs1205 and *HNF1A* rs1183910*, LEPR* rs4420065*, GCKR* rs1260326*, NLRP3* rs12239046*, IL1F10* rs6734238, *ASCL1* rs10745954, and ≥90% to detect OR ≥ 1.4 for *CRP* rs1800947 and *PPP1R3B* rs9987289 and *HNF4A* rs1800961 and *SALL1* rs10521222.

### Influence of *CRP* polymorphisms on CV events or subclinical atherosclerosis in patients with RA

We assessed the potential influence of *CRP* polymorphisms (rs1417938, rs1800947 and rs1205) on the risk of CV events or subclinical atherosclerosis in RA patients.

The linkage disequilibrium (LD) pattern of these 3 *CRP* polymorphisms obtained by HapMap Project phase I, II and III and Haploview (v.4.2) software and measured by r^2^ coefficient is displayed in Supplementary Fig. online.

After adjustment for potential confounder factors, no statistically significant differences were found when each *CRP* polymorphism was assessed independently and according to the presence/absence of CV events, carotid plaques and cIMT values (Table 2). Similarly, after adjustment for potential confounder factors, no statistically significant differences were detected when *CRP* polymorphisms were tested together conforming haplotypes and according to the presence/absence of CV events, carotid plaques and cIMT values (Table 3).

### Influence of *HNF1A, LEPR, GCKR, NLRP3, IL1F10, PPP1R3B, ASCL1, HNF4A* and *SALL1* polymorphisms on CV events or subclinical atherosclerosis in patients with RA

In addition, we evaluated the potential relationship between *HNF1A* rs1183910*, LEPR* rs4420065*, GCKR* rs1260326*, NLRP3* rs12239046*, IL1F10* rs6734238*, PPP1R3B* rs9987289*, ASCL1* rs10745954*, HNF4A* rs1800961 and *SALL1* rs10521222 polymorphisms and CV events or subclinical atherosclerosis in RA patients (Table 4). Accordingly, no significant differences were obtained when RA patients were stratified according to the presence/absence of CV events after adjustment for potential confounder factors (Table 4). It was also the case when RA patients were stratified according to the presence/absence of carotid plaques and the evaluation of cIMT after adjustment for potential confounder factors (Table 4).

### Influence of *CRP, HNF1A, LEPR, GCKR, NLRP3, IL1F10, PPP1R3B, ASCL1, HNF4A* and *SALL1* polymorphisms on CRP serum levels in patients with RA

Furthermore, we determined the potential influence of *CRP, HNF1A, LEPR, GCKR, NLRP3, IL1F10, PPP1R3B, ASCL1, HNF4A and SALL1* on CRP serum levels at RA diagnosis and also at the time of the carotid US study. In this regard, the mean ± SD of CRP serum levels at RA diagnosis in patients carrying the minor *CRP* rs1417938A allele was 12.66 ± 25.32 mg/l *versus* 9.80 ± 19.39 mg/l in those carrying the major *CRP* rs1417938T allele (p = 0.0002) (Supplementary Table S1). Additionally, the mean ± SD of CRP serum levels at the time of the carotid US study in patients carrying the minor *CRP* rs1417938A allele was 7.15 ± 19.16 mg/l *versus* 5.64 ± 14.56 mg/l in those carrying the major *CRP* rs1417938T allele (p = 0.029) (Supplementary Table S1). Moreover, the mean ± SD of CRP serum levels at the time of the carotid US study in patients carrying the minor *CRP* rs1205T allele was 5.02 ± 12.81 mg/l *versus* 6.71 ± 17.70 mg/l in those carrying the major *CRP* rs1205C allele (p = 0.018) (Supplementary Table S1). Consistent with these results, the mean ± SD of CRP serum levels at RA diagnosis in patients carrying the ACC haplotype (which harbors the minor *CRP* rs1417938A allele) was 14.08 ± 28.28 mg/l *versus* 10.23 ± 19.90 mg/l in those patients carrying the TCC haplotype, the most common haplotype found in our series (p = 0.0002) (Supplementary Table S2). Also, the mean ± SD of CRP serum levels at the time of the carotid US study in patients carrying the TCT haplotype (which harbors the minor *CRP* rs1205T allele) was 4.34 ± 7.75 mg/l *versus* 6.14 ± 16.46 mg/l in those patients carrying the TCC haplotype (p = 0.023) (Supplementary Table S2). However, no association between *HNF1A, LEPR, GCKR, NLRP3, IL1F10, PPP1R3B, ASCL1, HNF4A* and *SALL1* and both CRP serum levels at RA diagnosis and at the time of the carotid US study was disclosed (Supplementary Table S3).

## Discussion

Several studies have suggested that CRP has direct effects on the vessel wall promoting atherosclerosis^19^. Among them, CRP seems to stimulate the production of cellular adhesion molecules by vascular endothelial cells, facilitate the adhesion and migration of monocytes through the vessel wall, mediate the uptake of low-density lipoprotein cholesterol by macrophages and cause complement activation^19^.

An association between high-grade, chronic CRP elevation and subclinical atherosclerosis in patients with RA has been reported^11^. Additionally, a higher risk of CV events in patients with RA with chronic inflammation expressed by persistently increased CRP serum levels has been found^2^.

Since *CRP*, *HNF1A*, *LEPR*, *GCKR*, *NLRP3*, *IL1F10*, *PPP1R3B*, *ASCL1*, *HNF4A* and *SALL1* have been described as significant signals associated with elevated CRP serum levels in non-rheumatic Caucasians^12^, we set up a large-scale study to determine the potential influence of these 10 genetic variants on the development of atherosclerotic disease in patients with RA. Interestingly, our results disclosed a relationship between *CRP* gene and CRP serum levels at RA diagnosis and at the time of the carotid US study. However, we could not find an association between the elevated CRP serum level-related variants in non-rheumatic Caucasians and the presence of subclinical atherosclerosis or CV events in RA patients.The lack of evidence for the association between these polymorphisms and atherosclerosis in RA patients does not exclude the implication of CRP in the pathogenesis of this disease. This protein is produced as a part of an intricate acute phase response where several inflammatory factors, closely interrelated, are involved. Although we could not find an association of *IL6* gene polymorphisms with CV disease^20^, it is possible that complex interactions between elevated CRP serum level-related genes in non-rheumatic Caucasians and other genes implicated in the inflammation cascade may lead to up-regulation of CRP, promoting the progression of accelerated atherosclerosis in RA patients.

In summary, the results obtained in the present study do not confirm association between *CRP*, *HNF1A*, *LEPR*, *GCKR*, *NLRP3*, *IL1F10*, *PPP1R3B*, *ASCL1*, *HNF4A* and *SALL1* and CV disease in patients with RA.

## Additional Information

**How to cite this article**: López-Mejías, R. *et al.* Influence of elevated-CRP level-related polymorphisms in non-rheumatic Caucasians on the risk of subclinical atherosclerosis and cardiovascular disease in rheumatoid arthritis. *Sci. Rep.* **6**, 31979; doi: 10.1038/srep31979 (2016).

## Supplementary Material

Supplementary Information

## Figures and Tables

**Table 1:** Data on epidemiological and demographical features of the 2,313 patients with rheumatoid arthritis from Spain recruited in this work.

Variables	% (n/N)
Age at RA onset (years, mean ± SD)	51.0 ± 14.7
Follow-up time (years, mean ± SD)	11.6 ± 8.7
Women (%)	72.4
RF positive*	64.8 (1,265/1,952)
Anti-cyclic citrullinated peptide antibodies positive	58.7 (1,171/1,996)
Erosions	55.6 (1,086/1,954)
Extra-articular manifestations**	25.0 (448/1,789)
Cardiovascular risk factors	
Hypertension	37.6 (822/2,185)
Diabetes mellitus	12.1 (264/2,185)
Dyslipidemia	36.4 (796/2,185)
Obesity	21.7 (474/2,185)
Smoking habit	35.3 (772/2,185)
Cardiovascular events	16.7 (386/2,313)
Ischemic heart disease	7.8 (181/2,313)
Heart failure	5.7 (131/2,313)
Cerebrovascular accident	4.7 (108/2,313)
Peripheral arteriopathy	2.1 (49/2,313)

SD: Standard deviation; RF: rheumatoid factor. *At least two determinations were required. **As previously described^2^.

**Table 2:** Association between *CRP* polymorphisms according to the presence/absence of CV events or subclinical atherosclerosis in RA patients.

	Change	Presence/absence of CV events (n = 2,313)		Presence/absence of carotid plaques (n = 1,298)		cIMT (n = 1,298)
		P*	HR [95% CI]*	P**	OR [95% CI]**	P***
*CRP* rs1417938	T/A	0.26	1.18 [0.88–1.58]	0.24	0.87 [0.69–1.10]	0.63
*CRP* rs1800947	C/G	0.57	0.83 [0.43–1.58]	0.78	1.07 [0.66–1.75]	0.39
*CRP* rs1205	C/T	0.72	1.06 [0.78–1.43]	0.35	1.12 [0.88–1.42]	0.35

CV: cardiovascular; RA: rheumatoid arthritis; cIMT: carotid intima-media thickness; HR: hazard ratios; CI: confidence interval; OR: odds ratio. *Adjustment for gender, follow-up time, age at RA diagnosis and traditional CV risk factors by Cox regression. **Adjustment for gender, follow-up time, age at the time of ultrasonography study and traditional CV risk factors by logistic regression. ***Adjustment for gender, follow-up time, age at the time of ultrasonography study and traditional CV risk factors using analysis of covariance (ANCOVA).

**Table 3:** Results of *CRP* haplotype analysis according to the presence/absence of CV events or subclinical atherosclerosis in RA patients.

Haplotypes			Presence/absence of CV events		Presence/absence of carotid plaques		cIMT
**rs1417938**	**rs1800947**	**rs1205**	P*	HR [95% CI]*	P**	OR [95% CI]**	P***
T	C	C	—	1 (reference)	—	1 (reference)	—
A	C	C	0.21	1.19 [0.90–1.60]	0.21	1.14 [0.92–1.41]	0.54
T	C	T	0.37	0.86 [0.62–1.19]	0.78	0.95 [0.64–1.39]	0.41
A	C	T	0.72	0.93 [0.62–1.39]	0.60	1.15 [0.69–1.89]	0.60

CV: cardiovascular; RA: rheumatoid arthritis; cIMT: carotid intima-media thickness; HR: hazard ratios; CI: confidence interval; OR: odds ratio. *Adjustment for gender, follow-up time, age at RA diagnosis and traditional CV risk factors by Cox regression. **Adjustment for gender, follow-up time, age at the time of ultrasonography study and traditional CV risk factors by logistic regression. ***Adjustment for gender, follow-up time, age at the time of ultrasonography study and traditional CV risk factors using analysis of covariance (ANCOVA).

**Table 4:** Association between *HNF1A, LEPR, GCKR, NLRP3, IL1F10, PPP1R3B, ASCL1, HNF4A, SALL1* according to the presence/absence of CV events or subclinical atherosclerosis in RA patients.

	Change	Presence/absence of CV events (n = 2,313)		Presence/absence of carotid plaques (n = 1,298)		cIMT (n = 1,298)
		P*	HR[95% CI]*	P**	OR[95% CI]**	P***
*HNF1A* rs1183910	G/A	0.96	0.99[0.73–1.36]	0.55	0.93[0.73–1.18]	0.63
*LEPR* rs4420065	C/T	0.24	1.19[0.89–1.58]	0.57	0.94[0.75–1.18]	0.54
*GCKR* rs1260326	C/T	0.51	0.91[0.68–1.21]	0.36	1.11[0.89–1.38]	0.35
*NLRP3* rs12239046	C/T	0.72	0.95[0.71–1.27]	0.19	0.86[0.69–1.08]	0.57
*IL1F10* rs6734238	A/G	0.88	0.98[0.74–1.30]	0.17	1.17[0.94–1.46]	0.34
*PPP1R3B* rs9987289	G/A	0.55	0.84[0.46–1.52]	0.60	0.89[0.59–1.36]	0.19
*ASCL1* rs10745954	A/G	0.81	1.04[0.78–1.38]	0.67	0.95[0.76–1.19]	0.92
*HNF4A* rs1800961	C/T	0.32	1.51[0.67–3.43]	0.50	1.27[0.63–2.60]	0.72
*SALL1* rs10521222	C/T	0.97	1.01[0.53–1.91]	0.58	0.87[0.53–1.43]	0.75

CV: cardiovascular; RA: rheumatoid arthritis; cIMT: carotid intima-media thickness; HR: hazard ratios; CI: confidence interval; OR: odds ratio. *Adjustment for gender, follow-up time, age at RA diagnosis and traditional CV risk factors by Cox regression. **Adjustment for gender, follow-up time, age at the time of ultrasonography study and traditional CV risk factors by logistic regression. ***Adjustment for gender, follow-up time, age at the time of ultrasonography study and traditional CV risk factors using analysis of covariance (ANCOVA).
