# Supplementary material for: Influence of elevated-CRP level-related polymorphisms in non-rheumatic Caucasians on the risk of subclinical atherosclerosis and cardiovascular disease in rheumatoid arthritis
Source: Sci Rep. 2016 Aug 18;6:31979. doi: 10.1038/srep31979 (PMC4989194; doi:10.1038/srep31979)
Supplement: Supplementary Information [file srep31979-s1.pdf]

Influence of elevated-CRP level-related polymorphisms in non-rheumatic Caucasians on the risk of subclinical atherosclerosis and cardiovascular disease in rheumatoid arthritis

Raquel López-Mejías[1¥], Fernanda Genre[1¥], Sara Remuzgo-Martínez[1¥], Carlos González-Juanatey[2], Montserrat Robustillo-Villarino[3], Javier Llorca[4], Alfonso Corrales[1], Esther Vicente[5], José A. Miranda-Filloy[6], César Magro[7], Beatriz Tejera-Segura[8], Marco A. Ramírez Huaranga[9], Trinitario Pina[1], Ricardo Blanco[1], Juan J. Alegre-Sancho[3], Enrique Raya[7], Verónica Mijares[1], Begoña Ubilla[1], María D. Mínguez Sánchez[9], Carmen Gómez-Vaquero[10], Alejandro Balsa[11], Dora Pascual-Salcedo[11], Francisco J. López-Longo[12], Patricia Carreira[13], Isidoro González-Álvaro[5], Luis Rodríguez-Rodríguez[14], Benjamín Fernández-Gutiérrez[14], Iván Ferraz-Amaro[8], Santos Castañeda[5], Javier Martín[15§], and Miguel A. González-Gay[1, 16, 17§*]

Supplementary Table S1. Association between *CRP* polymorphisms and CRP serum levels in RA patients.

	Allele	CRP serum levels at RA diagnosis mean ± SD (mg/l) (n=1,662)	p	CRP serum levels at the time of carotid US study mean ± SD (mg/l) (n=1,193)	p
CRP rs1417938	T	9.80 ± 19.39	0.0002	5.64 ± 14.56	0.029
	A	12.66 ± 25.32		7.15 ± 19.16	
CRP rs1800947	C	10.95 ± 21.91	0.06	6.17 ± 16.33	0.83
	G	7.87 ± 1.01		5.85 ± 17.94	
CRP rs1205	C	11.44 ± 22.82	0.06	6.71 ± 17.70	0.018
	T	9.92 ± 19.43		5.02 ± 12.81	

CRP: C-reactive protein; RA: rheumatoid arthritis; SD: standard deviation; US: ultrasound.

Supplementary Table S2. Association between *CRP* haplotypes and CRP serum levels in RA patients.

Haplotypes			CRP serum levels at RA diagnosis mean ± SD (mg/l)	p	CRP serum levels at the time of carotid US study mean ± SD (mg/l)	p
**rs1417938**	**rs1800947**	**rs1205**				
T	C	C	10.23 ± 19.90	Ref.	6.14 ± 16.46	Ref.
A	C	C	14.08 ± 28.28	0.0002	7.73 ± 20.13	0.11
T	C	T	9.76 ± 20.18	0.64	4.34 ± 7.75	0.023
A	C	T	11.55 ± 21.11	0.26	6.32 ±19.22	0.89

CRP: C-reactive protein; RA: rheumatoid arthritis; SD: standard deviation; US: ultrasound.

Supplementary Table S3. Association between *HNF1A, LEPR, GCKR, NLRP3, IL1F10, PPP1R3B, ASCL1, HNF4A, SALL1* polymorphisms and CRP serum levels in RA patients.

	Allele	CRP serum levels at RA diagnosis mean ± SD (mg/l) (n=1,662)	p	CRP serum levels at the time of carotid US study mean ± SD (mg/l) (n=1,193)	p
HNF1A rs1183910	G	10.86 ± 21.33	0.39	6.43 ± 16.86	0.14
	A	10.19 ± 21.51		5.38 ± 14.63	
LEPR rs4420065	C	10.85 ± 21.79	0.52	6.41 ± 16.03	0.33
	T	10.37 ± 21.18		5.75 ± 16.82	
GCKR rs1260326	C	10.60 ± 21.15	0.64	5.91 ± 15.99	0.42
	T	10.94 ± 22.30		6.44 ± 16.73	
NLRP3 rs12239046	C	10.48 ± 20.37	0.55	6.35 ± 17.71	0.51
	T	10.93 ± 23.31		5.91 ± 13.99	
IL1F10 rs6734238	A	10.77 ± 21.54	0.94	6.07 ± 15.67	0.75
	G	10.82 ± 21.72		6.29 ± 17.43	
PPP1R3B rs9987289	G	10.79 ± 21.81	0.21	6.24 ± 16.32	0.96
	A	9.07 ± 18.58		6.17 ± 17.24	
ASCL1 rs10745954	A	10.62 ± 21.60	0.78	6.40 ± 16.85	0.41
	G	10.83 ± 20.62		5.85 ± 15.70	
HNF4A rs1800961	C	10.55 ± 21.09	0.48	6.32 ± 16.56	0.11
	T	11.95 ± 2.71		3.29 ± 5.14	
SALL1 rs10521222	C	10.77 ± 21.79	0.46	6.23 ± 16.57	0.97
	T	9.61 ± 19.29		6.17 ± 14.07	

CRP: C-reactive protein; RA: rheumatoid arthritis; SD: standard deviation; US: ultrasound.

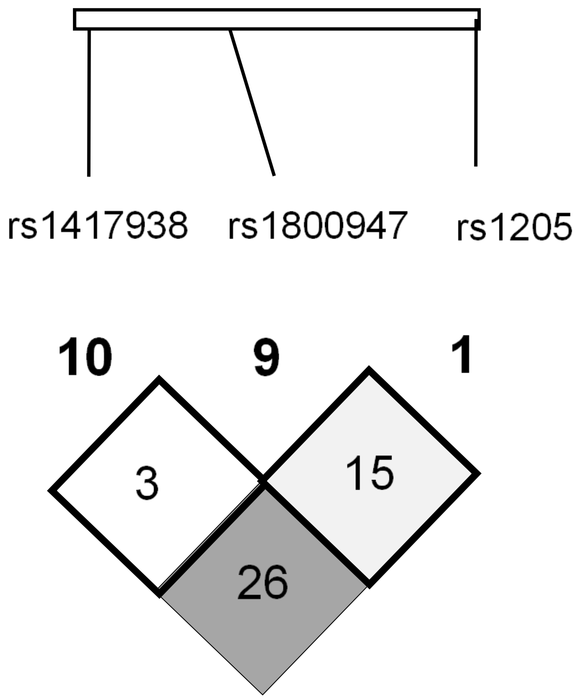

Supplementary Fig. Linkage disequilibrium (LD) pattern of the *CRP* polymorphisms (*CRP* rs1417938, *CRP* rs1800947, *CRP* rs1205) analyzed in our study measured by r^2 coefficient. Data obtained by HapMap Project phase I, II and III and Haploview (v.4.2) software. The LD between the *CRP* polymorphisms studied is shown in a scale from minimum (white) to maximum (black).
